# Longitudinal changes in body composition and water turnover in a world‐class male judo athlete: A case report

**DOI:** 10.14814/phy2.70965

**Published:** 2026-06-07

**Authors:** Narumasa Takubo, Emi Kondo, Akiko Uchizawa, Hiroaki Hiraoka, Katsuyuki Masuchi, Hirotaka Okada, Hiroyuki Sagayama

**Affiliations:** ^1^ Graduate School of Comprehensive Human Sciences University of Tsukuba Ibaraki Japan; ^2^ Department of Sports Sciences Osaka University of Health and Sport Sciences Osaka Japan; ^3^ Institute of Health and Sport Sciences University of Tsukuba Ibaraki Japan; ^4^ Department of Sports Science Japan Institute of Sports Sciences Tokyo Japan; ^5^ Japan Society for the Promotion of Science Tokyo Japan; ^6^ Advanced Research Initiative for Human High Performance (ARIHHP) University of Tsukuba Ibaraki Japan

**Keywords:** athletic performance, body water, hydration, longitudinal studies, men, weight loss

## Abstract

Limited longitudinal data on the physiology of world‐class athletes in weight‐category sports are currently available. This case report describes 3‐year longitudinal changes in body composition and water turnover (WT) in a world‐class male judo athlete during his second to fourth collegiate years. WT and total body water were assessed using the deuterium dilution method, with measurements conducted annually during these 3 years. Throughout the study period, even during periods of recovery from injury, the participant maintained a rigorous training load of 11 sessions/week by substituting intensive resistance training for judo. Between the second and third years, his body mass increased by 2.7 kg, with fat‐free mass (FFM) accounting for approximately three‐quarters of this gain (1.9 kg). Similar increases in body mass and FFM were observed between the second and fourth years. Seasonal fluctuations in WT accompanied these changes, reflecting varying training loads and competition schedules. This report provides a proof of concept for the high‐precision monitoring of world‐class athletes, thus offering valuable insights into body water dynamics and seasonal physiological variations for developing safer weight‐management strategies in judo and other combat sports.

## 1 INTRODUCTION

As judo is a weight‐class sport, judo athletes must pass the official weigh‐in before competitions. Consequently, many judo athletes experience weight loss before weigh‐in and regain weight through recovery in each competition (Štangar et al., 2022). These body mass variations are generally accompanied by changes in body composition and water turnover (WT), which are important factors affecting weight control and performance (Reale et al., 2017). WT represents the daily flux of water through the body, which is equivalent to water influx (water intake from all sources plus metabolic water production) and water efflux (total water loss) (Yamada et al., 2022). Silva et al. (2010, 2011) reported that reductions in total body water (TBW) leading to body composition changes are associated with decreased upper‐body power output and maximal forearm strength in judo athletes. Despite the effects of acute dehydration being well established, such as rapid weight loss through fluid restriction and dehydration before competitions (Bialowas et al., 2023; Štangar et al., 2022), there is a lack of longitudinal data regarding how WT and body composition in elite judo athletes change during different seasons, and how minor weight control can be facilitated by understanding daily WT and body composition (Reale et al., 2017). Although clarifying the daily WT in elite athletes can provide important insights for hydration management and the monitoring of body mass, researchers have mainly focused on short‐term interventions or general athlete populations (Sagayama et al., 2014). However, given the challenges associated with long‐term monitoring using high‐precision methods, data from world‐class athletes remain insufficient. These athletes represent a level of physiological adaptation that cannot be extrapolated from data obtained in general athlete populations, underscoring the need for direct, high‐precision monitoring at the elite level. In this case report, we address this gap by tracking a world‐class judo athlete throughout 3 years of collegiate life, while utilizing a gold‐standard stable isotope dilution method to measure WT. By documenting these longitudinal changes, we aimed to provide a proof of concept for high‐precision monitoring of world‐class athletes. Rather than serving as a generalizable strategic guide, this report highlights the complex interplay of physiological adaptations and WT during a critical phase of elite physical development.

## 2 METHODS

### 2.1 Participant

The participant was a 20‐year‐old world‐class male judo athlete in a university judo club at the study's start. At the time, he was transitioning to the senior international stage, establishing high stakes for his physical development. Training and performance status were tier 5, corresponding to World Class Level athletes (McKay et al., 2022). The participant placed first at two world‐level tournaments and second at a major international competition. Throughout the 3‐year tracking, the participant maintained high training volume for frequent international and national competitions. Excluding brief tapering before competitions, this training regimen was maintained year‐round. Typically, the participant completed 11 sessions/week (six judo, three running, and two resistance training sessions). Although the weekly session volume remained static, training intensity, internal load, and periodization varied dynamically across the macrocycle for continuous physiological adaptations. During injury‐related interruptions to judo training, the total volume was maintained by increasing the frequency of resistance training to eight sessions/week, focusing on muscle strengthening and rehabilitation. The participant demonstrated full adherence to the isotope dilution protocols and reported no excessive burden from the measurements throughout the study. This case report followed CARE guidelines (Gagnier et al., 2013).

### 2.2 Measurements

Three annual measurements were taken from the athlete's second to fourth collegiate years under weight‐stable conditions, intended to reflect his physiological baseline. While avoiding measurements during acute weight‐loss phases limits our insights into the immediate dynamics of competition preparation, it was necessary to prevent transient fluid shifts from confounding the longitudinal assessment of tissue accretion. To ensure that TBW/fat‐free mass (FFM) ratios were not substantially influenced by extreme dehydration or rapid weight loss protocols, all measurements were performed during periods of weight stability (Kondo et al., 2018; Sagayama et al., 2024). WT and TBW were measured using deuterium dilution. Urine or saliva samples were collected at baseline before stable isotope administration, 4 h after administration, in the morning and afternoon of the following day (day 1), and in the morning and afternoon of day 7. Urine samples were collected during the second and third collegiate years, and saliva during the fourth. The equivalence of TBW assessed from these fluids via deuterium dilution is well established (Sagayama et al., 2021). Prior to testing, fluid and food intake were controlled; the participant was instructed to fast overnight for at least 10 h after dinner and abstain from alcohol. The samples were analyzed using a stable isotope ratio mass spectrometry (Hydra 20‐20 Stable Isotope Mass Spectrometers; Sercon Ltd., Crewe, UK). WT was calculated from the 1‐week elimination rate of the stable isotope and deuterium dilution space (Nd × kd) (Yamada et al., 2022). Body mass was measured using a multi‐frequency bioelectrical impedance analyzer (MC‐780A‐N, TANITA Corp., Tokyo, Japan), in the morning under fasting conditions, following urination and defecation. TBW was determined using isotope dilution methods based from which FFM was calculated by dividing TBW by the constant 73.2% (FFM = TBW/0.732) (Pace & Rathbun, 1945). In our previous study, we confirmed the validity of this constant in athlete populations (Sagayama et al., 2020). Fat mass (FM) was calculated by subtracting FFM from body mass.

## 3 RESULTS

**FIGURE 1:**
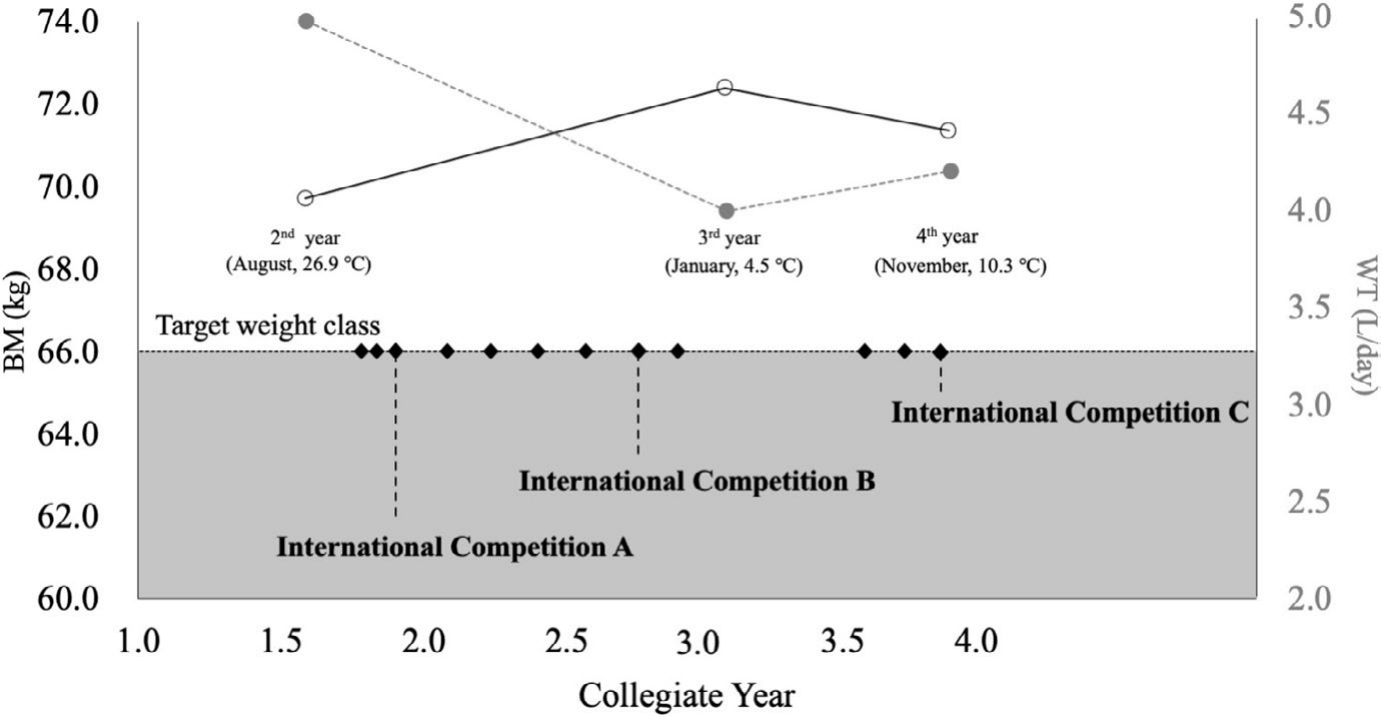
Variation in body mass and water turnover The open circles (◯) indicate BM throughout college life under non‐weight control conditions. WT is shown by gray‐filled circles (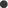) with dashed lines. The black diamonds (◆) indicate the dates of competition in this weight class. Although the participant successfully met the weigh‐in requirement (BM ≤66.0 kg) on these dates, these specific weigh‐in mass values were not measured or recorded as experimental data points for this study. Environmental temperatures (°C) are indicated for each measurement point. BM, body mass; WT, water turnover.

**FIGURE 2:**
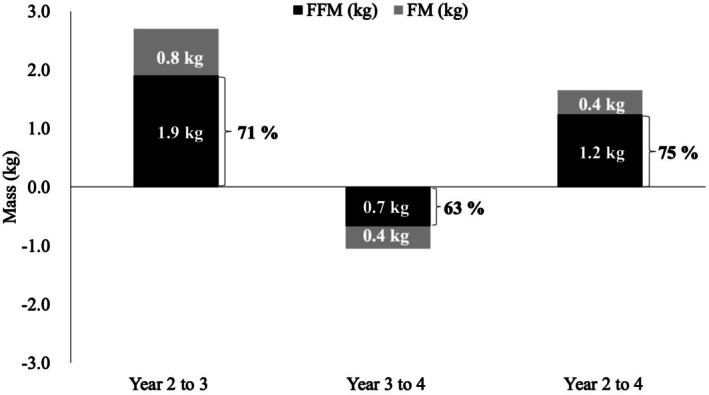
Variation in body mass, fat mass, and fat‐free mass The black squares (■) show FFM and gray squares (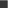) show FM. FFM, fat‐free mass; FM, fat mass.

Participant characteristics are summarized in Table 1. As shown in Figures 1 and 2, body mass and FFM increased from the second to the third collegiate year, followed by a slight decrease in the fourth year. The overall increase in body mass between the second and fourth years consisted primarily of FFM (approximately three‐quarters of the total body mass gain) (Figure 2). TBW followed a similar pattern to FFM. WT fluctuated, reaching its lowest point during the third collegiate year (Figure 1).

**TABLE 1:** Physical characteristics of the elite male judo athlete in this case report.

Collegiate year	2nd	3rd	4th
Age (years)	20	21	22
Measurement	2021 August	2023 January	2023 November
Body mass (kg)	69.7	72.4	71.4
Fat‐free mass (kg)	60.9	62.8	62.1
Fat mass (kg)	8.8	9.6	9.2
Total body water (kg)	44.6	46.0	45.5
Water turnover (L/day)	4.97	3.99	4.20

*Note*: Fat‐free mass was calculated from total body water, assuming an FFM hydration of 73.2%. Fat mass was calculated by subtracting fat‐free mass from body mass, and total body water and water turnover were measured using the stable isotope dilution method.

## 4 DISCUSSION

This case report was aimed at longitudinally tracking body composition and WT in a world‐class male judo athlete. Over 3‐year tracking period, the participant gained ~1.6 kg of body mass, with estimated FFM accounting for three‐quarters of this increase (Figures 1 and 2). However, since FFM was derived from TBW assuming a constant hydration fraction, this FFM‐predominant weight gain should be interpreted cautiously. This highly efficient weight gain contrasts with the 61.4% FFM accrual in young male judo athletes (Prieske et al., 2020). Consistent with previous reports (Casals et al., 2017), FM and FFM percentages are closely associated with performance level. World‐class male judo athletes typically maintain a body fat percentage <10% and possess significantly greater upper‐body muscle mass than non‐elite judo athletes (Franchini et al., 2005, 2011). Consequently, longitudinal FFM variations are a crucial performance determinant. This is exemplified by this athlete, who won 37 international matches between 2021 and 2024. Notably, 24 of these victories were achieved using strength‐dependent throwing techniques, which are characteristic of successful athletes from top‐tier judo nations (Ito et al., 2023). Thus, while isotope dilution provides only whole‐body averages and precludes confirming of regional hypertrophy without DXA or MRI, our athlete's ability to execute these throws was likely facilitated by overall FFM gains.

WT measurements were recorded during distinct seasonal periods (August 2021: 26.9°C, January 2023: 4.5°C, November 2023: 10.3°C; Figure 1). These observations suggest that WT variations are associated with environmental temperature, aligning with a large‐scale study reporting significantly higher temperatures in summer (29°C; 3.7 ± 1.0 L/day) than in spring (18°C; 3.0 ± 0.7 L/day) (Yamada et al., 2022). Additionally, physical activity drives WT (Yamada et al., 2022) partly due to metabolic water production—water generated internally during macronutrient oxidation—which increases proportionally to energy expenditure. Furthermore, since each gram of stored glycogen binds ~3–4 g of water (King et al., 2008), seasonal shifts in training volume and carbohydrate intake likely influence TBW and WT in athletes. Lacking direct daily fluid intake and sweat loss, these WT fluctuations require cautious interpretation. Nevertheless, these findings provide circumstantial evidence that seasonal factors may be associated with variations in the physiological baseline of an athlete. A lower WT in colder season suggests a reduced baseline fluid flux (e.g., lower daily sweat loss and fluid intake). Consequently, our athlete may have a narrower safety margin for utilizing sweat‐based rapid weight loss methods. Thus, approaching body mass reduction in winter with the same assumptions as in summer requires caution. These findings highlight the potential importance of considering seasonal variations in physiological parameters when planning individualized weight‐loss strategies.

Between the second and third years, the athlete's body mass increased by 2.7 kg (1.9 kg FFM), a trend continuing into the fourth year. Excluding brief pre‐competition tapers, he maintained 11 training sessions/week. This high baseline training may have contributed to sustaining muscle mass development. Notably, during the two injury‐related interruptions to the Judo Randori training for 4 months, the athlete's number of weekly sessions remained constant. To compensate for his inability to perform judo, he frequently underwent resistance training. However, the specific resistance training protocols (e.g., exact exercises, sets, and repetitions) were not recorded. By primarily performing Uchikomi and resistance training, athletes may help maintain or even enhance muscle mass and strength during injury. From our athlete's perspective, the alternative resistance training performed during the injury period was subjectively effective in maintaining his overall conditioning and muscle strength. Franchini et al. (2015) demonstrated that an 18‐week periodization program for judo and resistance training in judo athletes increases body mass without increasing fat percentage. Similarly, our athlete's alternative training successfully maintained conditioning and increased FFM. Furthermore, repeated cycles of pre‐competition weight loss and regain may prevent fat gain (Bagot et al., 2023), potentially increasing body mass via FFM.

Body water stored in muscles is expected to increase with increasing FFM; thus, TBW may increase as muscle mass develops and glycogen storage expands. FFM hydration remains relatively stable at ~73% (Wang et al., 2000), although intensive training could influence the distribution of fluids. For example, Ribeiro et al. (2014) reported that 16 weeks of hypertrophy‐oriented resistance training raises intracellular water levels. Herein, TBW increased with FFM, reflecting long‐term training and intensive resistance sessions during injury rehabilitation (up to eight sessions/week). These physiological changes may have direct implications for the athlete's weight‐management strategy. To comply with official weigh‐ins, our athlete typically reduced his body mass by 3–4 kg a few days before weigh‐in due to rapid weight loss methods (fluid restriction, fasting, sauna bathing, and wearing plastic clothing) (Bialowas et al., 2023; Štangar et al., 2022). However, achievable dehydration varies seasonally. Consequently, monitoring WT is valuable for planning individualized dehydration strategies. Furthermore, appropriate post‐weigh‐in rehydration strategies are critical, as optimal performance cannot be achieved without maintaining an adequate hydration status.

Despite these findings, we must note the limitation that this is a single‐case study; thus, results may not directly generalize to broader adult athletic populations. A major strength, however, is the 3‐year longitudinal assessment using the gold‐standard isotope dilution method. When utilizing this technique, fundamental assumptions, such as the rapid equilibration of isotopes and corrections for non‐aqueous hydrogen exchange, must be acknowledged (Sagayama et al., 2016). Despite this robust methodology, the lack of dietary and fluid records means that the causes of WT fluctuations and body composition maintenance during injury periods remaining speculative. Additionally, given judo's year‐round competition schedule, a single annual measurement prevents comparison of routine training with acute pre‐competition states. Furthermore, the mathematical dependence between TBW and FFM is a critical methodological limitation. Because FFM is derived from TBW using a fixed hydration constant, acute fluctuations in hydration status—which are prevalent in judo due to frequent fluid manipulation—could be erroneously reflected as muscle mass changes. Consequently, the observed longitudinal gains in FFM should be interpreted as estimated values rather than independently measured tissue accretion.

In conclusion, the collegiate weight gain of this elite athlete was characterized by a predominant increase in FFM rather than FM. Observed seasonal WT shifts indicate a non‐static physiological baseline for fluid fluctuation, pointing toward a need for more cautious weight‐loss protocols in winter. These findings offer valuable insight into body water dynamics for safer weight‐management strategies in adult male judo athletes. Future studies should incorporate more frequent assessment time points for top‐tier athletes and expand the research scope to include different competitive levels, beyond Tier 5. Similar longitudinal data on body water dynamics in adult female judo athletes must be accumulated.

## AUTHOR CONTRIBUTIONS

**Narumasa Takubo:** Conceptualization; data curation; formal analysis; investigation; methodology. **Emi Kondo:** Conceptualization; data curation; formal analysis; funding acquisition; investigation; methodology; project administration; resources; supervision; visualization. **Akiko Uchizawa:** Data curation; investigation; methodology; resources; supervision. **Hiroaki Hiraoka:** Conceptualization; data curation; investigation; methodology. **Katsuyuki Masuchi:** Investigation; methodology; resources. **Hirotaka Okada:** Data curation; funding acquisition; investigation; methodology; resources. **Hiroyuki Sagayama:** Conceptualization; data curation; formal analysis; funding acquisition; investigation; methodology; project administration; resources; software; supervision; validation; visualization.

## FUNDING INFORMATION

This study was supported by the Japan Society for the Promotion of Science KAKENHI for EK (21J00492) and for HS (23KK0177).

## CONFLICT OF INTEREST STATEMENT

The authors declare no conflict of interest.

## ETHICS STATEMENT

This study was conducted in accordance with the Declaration of Helsinki and was approved by the Institutional Review Board of the University of Tsukuba, Institute of Health and Sport Sciences (019‐32, 021‐85). The participant provided written informed consent. Informed consent for the publication of this case report was also obtained from the participant.

## Supporting information


Data S1.

## Data Availability

The datasets analyzed during this study are available from the corresponding author on reasonable request.
