# Supplementary material for: Longitudinal changes in body composition and water turnover in a world‐class male judo athlete: A case report
Source: Physiol Rep. 2026 Jun 7;14(11):e70965. doi: 10.14814/phy2.70965 (PMC13243879; doi:10.14814/phy2.70965)
Supplement: Supplementary file 1 — Data S1. [file PHY2-14-e70965-s001.pdf]

CARE Checklist of information to include when writing a case report

CARE case report guidelines

(cc) BY-NC-ND

Topic	Item	Checklist item description	Reported on Line
Title	1	The diagnosis or intervention of primary focus followed by the words "case report"	1-2
Key Words	2	2 to 5 key words that identify diagnoses or interventions in this case report, including "case report"	41
Abstract (no references)	3a	Introduction: What is unique about this case and what does it add to the scientific literature?	26-28, 36-39
	3b	Main symptoms and/or important clinical findings	32-35
	3c	The main diagnoses, therapeutic interventions, and outcomes	30-35
	3d	Conclusion—What is the main "take-away" lesson(s) from this case?	36-39
Introduction	4	One or two paragraphs summarizing why this case is unique (**may include references**)	50-66
Patient Information	5a	De-identified patient specific information.	69-70
	5b	Primary concerns and symptoms of the patient.	70-71
	5c	Medical, family, and psycho-social history including relevant genetic information	N/A
	5d	Relevant past interventions with outcomes	70
Clinical Findings	6	Describe significant physical examination (PE) and important clinical findings.	112-117
Timeline	7	Historical and current information from this episode of care organized as a timeline	Figure 1 and 2
Diagnostic Assessment	8a	Diagnostic testing (such as PE, laboratory testing, imaging, surveys).	88-110
	8b	Diagnostic challenges (such as access to testing, financial, or cultural)	89-92
	8c	Diagnosis (including other diagnoses considered)	N/A
	8d	Prognosis (such as staging in oncology) where applicable	N/A
Therapeutic Intervention	9a	Types of therapeutic intervention (such as pharmacologic, surgical, preventive, self-care)	78-80
	9b	Administration of therapeutic intervention (such as dosage, strength, duration)	75-80
	9c	Changes in therapeutic intervention (with rationale)	78-80, 154-155
Follow-up and Outcomes	10a	Clinician and patient-assessed outcomes (if available)	158-159
	10b	Important follow-up diagnostic and other test results	112-117
	10c	Intervention adherence and tolerability (How was this assessed?)	82-84
	10d	Adverse and unanticipated events	N/A
Discussion	11a	A scientific discussion of the strengths AND limitations associated with this case report	178-193
	11b	Discussion of the relevant medical literature **with references**.	124-128, 135-141
	11c	The scientific rationale for any conclusions (including assessment of possible causes)	135-147, 188-193
	11d	The primary "take-away" lessons of this case report (without references) in a one paragraph conclusion	194-199
Patient Perspective	12	The patient should share their perspective in one to two paragraphs on the treatment(s) they received	158-159
Informed Consent	13	Did the patient give informed consent? Please provide if requested	Yes ☒ No ☐
